# Oldest record of Machimosaurini (Thalattosuchia, Teleosauroidea): teeth and scavenging traces from the Middle Jurassic (Bajocian) of Switzerland

**DOI:** 10.1098/rsos.240071

**Published:** 2024-04-10

**Authors:** Torsten M. Scheyer, Michela M. Johnson, Dylan Bastiaans, Feiko Miedema, Erin E. Maxwell, Christian Klug

**Affiliations:** ^1^ Paläontologisches Institut, Universität Zürich, Zürich 8006, Switzerland; ^2^ Staatliches Museum für Naturkunde Stuttgart, Stuttgart 70191, Germany; ^3^ Naturkundemuseum Bamberg, Bamberg 96047, Germany

**Keywords:** Middle Jurassic, Teleosauroidea, Machimosaurini, Hauptrogenstein Formation, scavenging, feeding traces

## Abstract

The Jurassic period was a time of major diversification for Mesozoic marine reptiles, including Ichthyosauria, Plesiosauria and thalattosuchian Crocodylomorpha. The latter originated in the Early Jurassic and thrived during the Late Jurassic. Unfortunately, the Middle Jurassic, a crucial time in their evolution, has a poor fossil record. Here, we document the first evidence of macrophagous/durophagous Machimosaurini-tribe teleosauroid thalattosuchians from the late Bajocian (*ca* 169 Ma) in the form of three robust tooth crowns with conical blunt shapes and anastomosed pattern of thick enamel ridges towards the apex, associated with the skeleton of a large ichthyosaur lacking preserved tooth crowns. The tooth crowns were found on the posterior section of the lower jaw (left angular), a lacrimal and the axis neural arch of the ichthyosaur. In addition, some of the distal sections of the posterior dorsal ribs of the ichthyosaur skeleton exhibit rounded bite marks and some elongated furrows that fit in size and shape with the Machimosaurini teeth. These marks, together with the absence of healing in the rib bone are interpreted here as the indicators of peri- to post-mortem scavenging by a Machimosaurini teleosauroid after the large ichthyosaur carcass settled on the floor of a shallow ocean.

## 1. Introduction

Many Mesozoic marine reptile clades have their origins in the Triassic period, but for some of the most diverse and long-lived groups, an important radiation happened during the Early Jurassic (*ca* 201–145 Ma). Ichthyosaurs and plesiosaurs, which first appeared in the Early and Late Triassic, respectively [1,2], and thalattosuchian crocodylomorphs, which originated in the early Lower Jurassic (Hettangian–Sinemurian [3]), diversified and reached a diversity peak during the Toarcian [4]. For the Middle Jurassic, however, species diversity and rock record analyses indicate a drastic decline in the richness of marine reptile species, especially for shallow marine inhabitants during the Aalenian to Bajocian (*ca* 174–168 Ma). Diversity levels recovered only in the late Middle Jurassic (Bathonian/Callovian), a phenomenon linked to sea-level change, general availability of marine fossiliferous formations, and the effect of highly productive Lagerstätten [5–7]. Thus, early Middle Jurassic reptiles constitute rare finds, with only a few valid species described globally (e.g. [8–10]).

The uniquely marine crocodylomorph clade Thalattosuchia comprises two groups, Metriorhynchoidea and Teleosauroidea [11]. Following the Toarcian, their record is characterized by Aalenian, Bathonian and Oxfordian materials, with five of the six Bathonian species belonging to Machimosauridae, a speciose clade inhabiting the western part of the Tethys Ocean [12]. Within Machimosauridae, certain taxa show a distinctly robust jaw morphology and blunt tooth shape that make them different from other machimosaurids having a specialized macrophagous/durophagous diet [13–15]. These taxa constitute the Machimosaurini tribe within Machimosauridae. *Yvridiosuchus boutilieri* [16] is the oldest Machimosaurini-tribe teleosauroid, appearing in the Bathonian, and the group gets more firmly established based on the record of *Lemmysuchus obtusidens* [17] from the UK and France in the Callovian. The Machimosaurini record extends to the Kimmeridgian (Late Jurassic), except for *Machimosaurus rex,* the largest machimosaurine teleosauroid from the early Lower Cretaceous of Tunisia [12,18]. It is noteworthy that the appearance of the macrophagous/durophagous morphotype in crocodylomorphs coincides with the appearance of large macropredatory pliosaurs in the Bajocian of Germany, France and Switzerland [9,19].

The purpose here is to describe three tooth crowns from the Bajocian, which constitute the oldest record of the Machimosaurini tribe. These teeth are considered a rare find indeed, as they were recovered in association with the skeletal remains of a new Middle Jurassic ichthyosaur [10]. Finally, the bite marks on the ichthyosaur skeleton are identified as potential scavenging traces left by the teeth of the Machimosaurini.

## 2. Material and methods

### 2.1. Material

**Figure 1.**
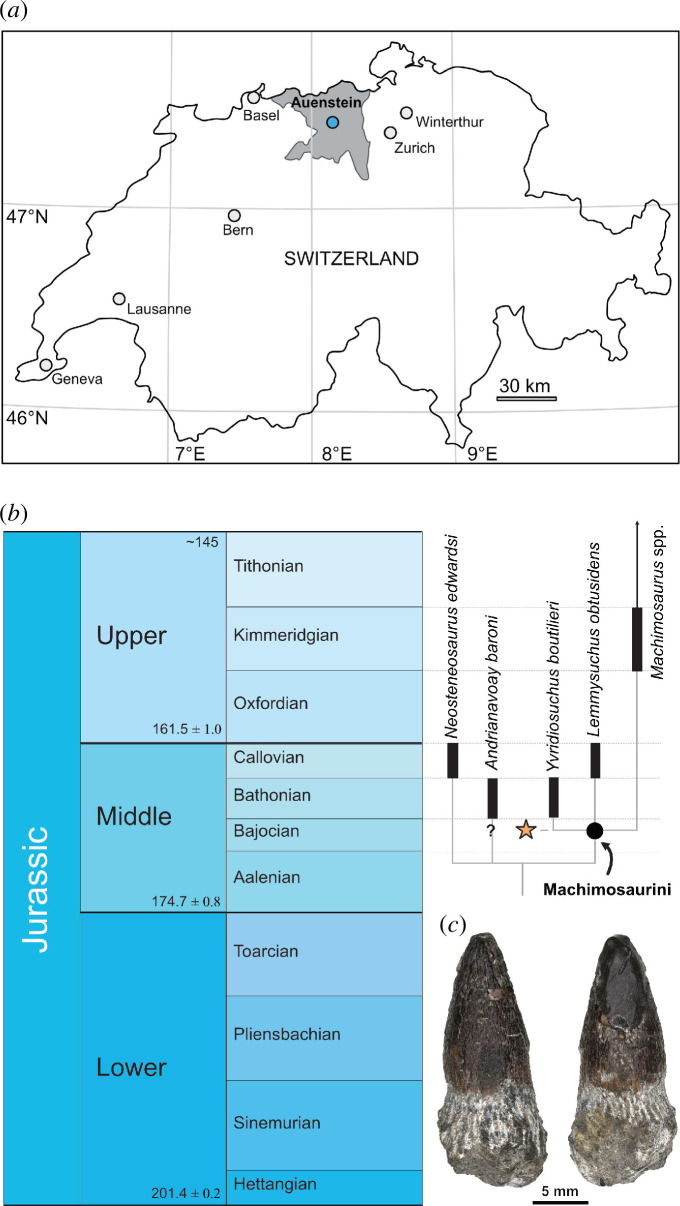
Finding location and phylogenetic framework of Swiss Machimosaurini tooth crowns. (*a*) Map of Switzerland with Canton of Aargau (in grey) and location of Auenstein indicated. (*b*) Time scale and abbreviated phylogeny based on Johnson *et al*. [12] with hypothesized position of new Machimosaurini indet. indicated by an orange star. (*c*) Isolated larger tooth crown (PIMUZ A/III 5281a).

The ichthyosaur skeleton (PIMUZ A/III 5279 [10]) and the three Machimosaurini teeth (two larger tooth crowns, PIMUZ A/III 5281a and PIMUZ A/III 5281b, associated with a lacrimal and the left angular of the ichthyosaur skeleton, respectively; PIMUZ A/III 5281c, a smaller tooth crown associated with the axis neural arch) were found in the Oberegg quarry, Auenstein, Canton Aargau, Switzerland (figure 1*a*). They were recovered from the Lower Acuminata beds of the Hauptrogenstein Formation (*subfurcatum/niortense* zone), which are late Bajocian (figure 1*b*) in age [20]. The original association of the teeth and the ichthyosaur skeleton was kept intact so that the teeth were still attached to the bones by a thin layer of limestone matrix, an exception being the third tooth, which was isolated to facilitate the examination of its morphology.

**Figure 2.**
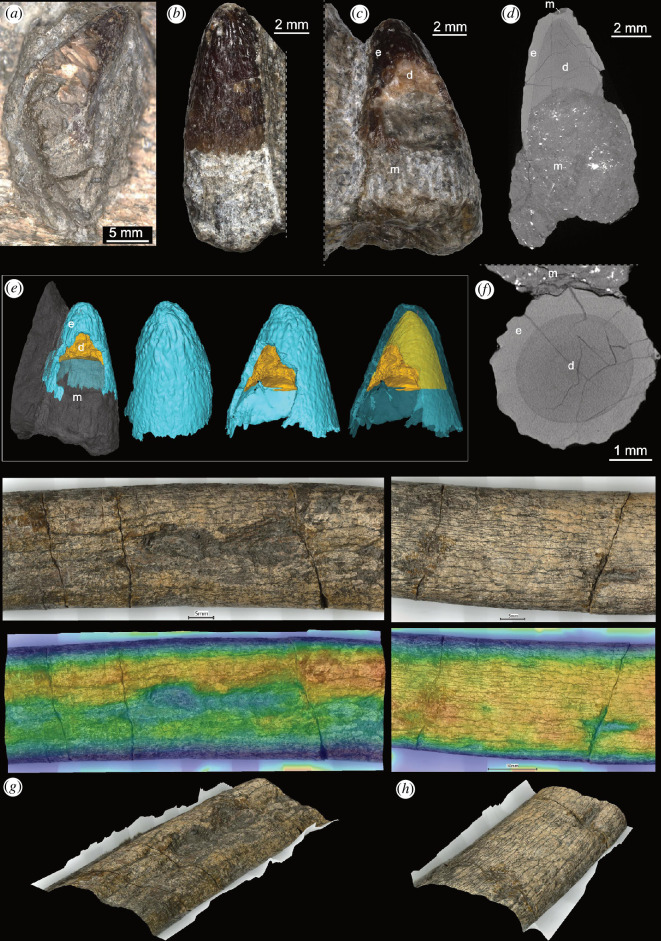
Photographs, CT scan and three-dimensional images of Swiss Machimosaurini tooth crowns and selected scavenging traces on the ichthyosaur ribs (PIMUZ A/III 5279). (*a*) Tooth crown PIMUZ A/III 5281b on left angular. (*b*,*c*) Tooth crown PIMUZ A/III 5281c on the axis neural arch. (*d*,*f*) CT scan images showing longitudinal and horizontal sections of PIMUZ A/III 5281c. (*e*) Three-dimensional model of PIMUZ A/III 5281c in different views, not to scale. (*g*,*h*) Normal, false colour and angled views of the ribs showing bite marks. Abbreviations: d, dentine; e, enamel; m, sediment matrix.

### 2.2. Methods

The documentation of the teeth and the bite marks on the ichthyosaur ribs was performed using a Keyence vhx-7000 digital microscope. In addition, the smaller of the tooth crowns (PIMUZ A/III 5281c) was computed tomography (CT)-scanned in-house at the University of Zurich with a Nikon XTH 225 ST CT scanner. The CT scan was taken with a voltage of 141 kV and a current of 206 µA, yielding a voxel size of 0.01027787 mm, with no ﬁlter used. The reconstruction of the digital stack (originally 3142 rotational projections, two frames, 250.0 ms exposure and 2000 tif slices in stack) for virtual three-dimensional reconstruction was done using MIMICS Innovation Suite 25.0 (Materialise NV, Leuven, Belgium).

### 2.3. Abbreviations

PIMUZ collections of the Department of Paleontology, University of Zurich, Zurich, Switzerland.

## 3. Results

### 3.1. Systematic palaeontology

Crocodylomorpha Walker, 1968 ([21] *sensu* [22])

Thalattosuchia Fraas, 1901 ([23] *sensu* [24])

Teleosauroidea Geoffroy Saint-Hilaire, 1831 ([25] *sensu* [24])

Machimosauridae Johnson *et al*., 2020 ([12] *sensu* [26])

Machimosaurinae Johnson *et al*., 2020 ([12] *sensu* [26])

Tribe Machimosaurini Jouve *et al*., 2016 ([27])

Machimosaurini indet. (figures 1 and 2)

### 3.2. Anatomical description of tooth crowns

All teeth exhibit a wide conical crown shape, which are blunt and robust and show neither carinae or labiolingual (= mediolateral) compression nor identifiable true or false denticles (compared to [28,29]). An anastomosed pattern of enamel ridges extends apicobasally over the whole crown. There appears to be no curvature at the apices. All crowns also experienced some local damage to the enamel and dentine; however, whether this damage occurred during the animal’s life, fossilization or preparation cannot be elucidated. The enamel thickness increases apically, although, without additional CT scanning, the boundary between the pulp cavity and dentine is difficult to discern in the larger teeth. In the two larger specimens, the dentine infilling is visible due to breaks in the enamel caps in both cases.

PIMUZ A/III 5281a (figure 1*c*) is a larger tooth crown, 15.53 mm long and up to 8.92 mm wide. About one-third of the apex of the crown was damaged and needed to be stabilized with a dark synthetic resin. Otherwise, the crown appears mostly complete with the apex having experienced slight erosion. The enamel ridges appear to be shallower at the base of the tooth crown to become more distinct, higher and more strongly branching towards the apex. Parallel marks in the sediment matrix proximal to the crown are identified as preparation traces left by pneumatic tools and are not part of any root tissue. PIMUZ A/III 5281b (figure 2) is very similar in proportion, being 15.41 mm high as preserved and 9.83 mm wide. The apex is complete without showing any damage at the tip or any worn facets. In PIMUZ A/III 5281c (figure 2), the crown is 9.08 mm high as preserved and 6.82 mm wide. The internal pulp cavity is restricted to about the lower half of the preserved crown, with dentine filling the upper half. The thickness of the outer enamel layer increases from the base of the crown to the apex, reaching a thickness of 0.5–0.65 mm. Apically, the enamel is locally damaged, leaving behind a shallow depression filled with sediment. The enamel ridges on the apex of the tooth appear to have an anastomosed (= wrinkled) texture, but they are not closely situated to one another and are more knobbly and ‘chaotic’/disorganized compared with other machimosaurins such as *Yvridiosuchus boutilieri*, *Lemmysuchus obtusidens* and *Machimosaurus* spp. [12,29–31]. The apicobasal enamel ridges are not well developed but appear to extend parallel from the perceived crown base to approximately three-quarters of the entire tooth. The enamel–dentine junction is smooth rather than scalloped, such that the ridges are constituted entirely by thickening of the enamel layer.

### 3.3. Bone modifications on the ichthyosaur ribs

Several ribs in the posterior trunk region of the ichthyosaur skeleton PIMUZ A/III 5279 show surficial marks on the bone cortex and upper parts of the cancellous interior (figure 2). These bone modifications are localized, often in rows and sometimes appear on opposite sides of the ribs. Furthermore, these modifications can be identified as furrows, pits and deeper punctures, which led to the collapse of the rib cortex [32] but did not pierce the ribs completely. The collapse of the rib cortex also argues against chemical or parasitic bone modification (e.g. [33]). The pitted and punctured rib bone also indicates that the tooth causing these modifications was of wide and blunt shape, likely lacking carinae [32]. There is no indication of healing or calluses forming around the marks.

## 4. Discussion and conclusions

The three tooth crowns are identified as those belonging to Machimosaurini indeterminate due to their blunt and wide crown shape, the anastomosed (‘wrinkled’) pattern of enamel ridges on the tooth apex and the absence of scalloping of the enamel–dentine junction underlying the enamel ridges. These three major dental characteristics of the group [12,29] set them apart from ichthyosaur and plesiosaur/pliosaur teeth. In these other marine reptile groups, apicobasal enamel ridges remain usually separate or show few anastomosing junctions [9,34–37]; in ichthyosaurs in particular, enamel ridges are formed through scalloping of the enamel–dentine junction rather than solely through thickening of the enamel layer [38]. The thickened enamel cap (more than 0.5 mm) of the crown, underlying massive dentine patch with little pulp cavity expansion towards the apex and overall tooth robustness, can further be interpreted as indicative of a macrophagous or durophagous diet [34].

Besides being blunt and robust in shape, the new Swiss specimens show round cross-sections with no mediolateral compression and short and often branching enamel ridges along the lower half of the crown, rather than longer parallel ones. The teeth of early diverging Machimosaurini such as *Lemmysuchus obtusidens* and *Yvridiosuchus boutilieri*, while also being large and robust and lacking mediolateral compression, are typically carinated and their crowns showing apicobasal curvature and denticulation on their mostly parallel-trending enamel ridges [28,30,31]. Round cross-sections and lack of carinae have been noted for some machimosaurine teeth as well, as seen in specimens pertaining to Machimosaurini indet. from the Corallian Group, UK [35, fig. 9], but these teeth are apicobasally curved with more ordered enamel ridges along the non-apical crown.

The lack of apparent tooth crown curvature in the new Swiss specimens could indicate that either (i) there simply was no curvature in any of these teeth or (ii) the teeth come from the posterior part of the tooth row in which teeth might be less curved compared to anterior teeth (as in *Yvridiosuchus* [30]). If the former would be the case, this together with the ‘irregular’ anastomosing pattern of the enamel ridges, the shorter branching enamel ridges, and the lack of carinae and denticulation of these Bajocian specimens might hint at the presence of a new, potentially early diverging taxon within Machimosaurini. Given the scarcity of the material, however, this remains speculative for now, while at the same time hinting at the hitherto untapped potential of filling the gaps in marine crocodyliform evolution, particularly in the Middle Jurassic.

The bone modifications found on the ichthyosaur ribs likely appeared peri- or post-mortem as bone remodelling and healing processes are absent. The large size of the ichthyosaur and the inferred thick layer of soft tissue overlying the ribs make scavenging a more plausible source for these traces rather than predation. Taphonomic interpretation suggests that the carcass lay exposed on the seafloor for a prolonged period prior to burial [10]. Furthermore, only posterior thoracic ribs bear the bite marks, which suggests that the scavenging machimosaurin(s) accessed the tissues from behind like many other scavengers today [39,40]. Despite the tooth crowns being found more anterior on the ichthyosaur skeleton and not in direct vicinity of the traces on the ribs, a close fit of tooth crown morphology with the shape of the bone modifications indicates that the latter are consistent with scavenging traces (e.g. [41]) of a small- to medium-sized macrophagous Machimosaurini thalattosuchian crocodyliform that co-inhabited the marine environment in the Bajocian.

## Data Availability

All the new Swiss fossils mentioned in the text are officially accessioned in the collections of the Department of Paleontology, University of Zurich, and can be studied upon request. The CT data set and the 3D surface model (in .ply format) are provided on Morphosource repository project ID: 000593818 [42].
